# Traffic Estimation for Large Urban Road Network with High Missing Data Ratio

**DOI:** 10.3390/s19122813

**Published:** 2019-06-24

**Authors:** Kennedy John Offor, Lubos Vaci, Lyudmila S. Mihaylova

**Affiliations:** 1 Department of Automatic Control and Systems Engineering, University of Sheffield, Sheffield S1 3JD, UK; l.s.mihaylova@sheffield.ac.uk; 2 Department of Computer Science, University of Sheffield, Sheffield S1 4DP, UK; l.vaci@sheffield.ac.uk

**Keywords:** particle filtering, road traffic, state estimation, Bayesian inference, Kriging, missing data imputation

## Abstract

Intelligent transportation systems require the knowledge of current and forecasted traffic states for effective control of road networks. The actual traffic state has to be estimated as the existing sensors does not capture the needed state. Sensor measurements often contain missing or incomplete data as a result of communication issues, faulty sensors or cost leading to incomplete monitoring of the entire road network. This missing data poses challenges to traffic estimation approaches. In this work, a robust spatio-temporal traffic imputation approach capable of withstanding high missing data rate is presented. A particle based approach with Kriging interpolation is proposed. The performance of the particle based Kriging interpolation for different missing data ratios was investigated for a large road network comprising 1000 segments. Results indicate that the effect of missing data in a large road network can be mitigated by the Kriging interpolation within the particle filter framework.

## 1. Introduction

Intelligent Transportation Systems (ITS) require accurate knowledge of traffic states for effective traffic monitoring and control. Traffic states are usually estimated from noisy sensor measurements [1] using various approaches, which can typically be subdivided into model based approaches, data driven approaches or streaming-data-driven [2]. An overview of the different modelling methodologies is given in Refs. [3,4]. These modelling methodologies include microscopic, macroscopic and mesoscopic approaches. Microscopic traffic models [3,4,5,6] describe the motion of each individual vehicle with a high level of detail. Hao et al. [7,8] recently proposed a model predictive control (MPC) termed urban cell transmission model (UCTM) for optimal switching of traffic lights at intersections based on the predicted traffic.

In macroscopic models [9,10], traffic state is represented by aggregating behaviour of the traffic, usually in terms of the average speed and the average density over a given period of time. Mesoscopic models [3] employ some features of microscopic and macroscopic approaches by utilising varying levels/degrees of detail to model traffic behaviour. This is achieved by modelling some locations with aggregated measurements as in macroscopic and the remaining locations are modelled down to the details of individual vehicles as is done in the case of microscopic.

Macroscopic models are sufficient to produce acceptable estimation accuracy when compared to the computational overhead of the microscopic models. Hence, they are the preferred choice for most practical purposes such as traffic control/management, road pricing and changes in infrastructure. Most traffic estimation approaches are model based [2], while the new trend is to develop data driven approaches [11].

Data driven methods rely on historical data or streaming/real-time data. Within the last decade, there has been growing interest in applying Kriging to traffic state prediction; for directional traffic volume using global position system (GPS) data [12], annual average traffic count interpolation using origin-destination data [13], estimating annual average daily traffic [14,15], traffic volume prediction [16,17], and traffic volume imputation [18].

Kriging is one of the most flexible data driven methods originally employed in geo-statistics or spatio-temporal analysis. Kriging exploits the spatial dependence (either covariance or variogram) using a weighted sum of observed data points to interpolate the values at locations of interest. It was initially used for copper mining by Krige [19] and was developed further by Matheron [20]. Since then, it has been applied in other fields such as spatial analysis and computer experiments. In the recent times, the method has been applied in traffic prediction [12].

One of the major challenges faced in traffic prediction is the issue of missing or sparse data. Traffic measurements are generally captured with different types of sensors and transmitted through communication infrastructure for processing and utilisation. These infrastructures are subject to failure and malfunction occasionally leading to incomplete/missing data, sometimes more than 40% [21]. The problem of sparse data is caused by the high cost of installing and managing traffic measurement devices making them impractical to cover all locations needed for effective observation of the full road network.

To address these challenges, researchers resorted to various methods and approaches such as missing data imputation [22], compressive sensing and historical averages [23], and Kriging interpolation [16]. In Ref. [24], a particle filter (PF) with the stochastic compositional model (SCM) is developed to estimate traffic states in motorways. Boundary measurements (inflow and outflow) were used to estimate the traffic state within the segments. The study reported that missing boundary measurements affected the estimated accuracy. A solution to the problem of missing data in particle filter measurement update was proposed in our previous work [25]. Here Kriging methods were used to interpolate the missing data which is subsequently used for the computation of the PF likelihood for traffic state estimation.

This work extends the approach of [25] for a larger road network of several kilometres with 1000 segments under the influence of missing data and/or sensor failure. The task of training such large segments would be resource intensive, hence the reduced measurement space, proposed in Ref. [26], was used to select the most influential segments in the road network. The drawback of Ref. [26] is that the most influential segments are selected based on only available measurements without considering any missing values. This would mean that if some interconnected segments have missing data, they would not be used leading to information loss. To address this, we propose a new approach, that combines the benefits of both approaches ([25,26]) in a single framework. First, the most probable segments are selected using the column based matrix decomposition (CBMD). Second, when there are missing measurements in the segments, Kriging is used to estimate the measurements before the measurement update step (see Section 5.3).

The present work differs from Refs. [12,16] in the following ways. Whereas [12,16] employed Kriging for predicting vehicular speed, our approach used Kriging to estimate missing vehicular flow and speed measurements which is then applied to the computation of measurement update step for particle filter estimation. In addition, our approach combines speed and vehicular flow estimation unlike the former which predicted only speed. The approaches used in Refs. [12,16] are similar, the only difference being that the former proposes the use of alternate distance metric called Approximate Road Distance Network (ARDN) to replace the Euclidean distance metric.

The contribution of this work is twofold: (i) A robust spatio-temporal traffic imputation that can withstand higher missing data proportion via information exchange between correlated segments. (ii) the use of Kriging and particle filter to address the effects of high missing/sparse data. A Column based matrix decomposition is used to select most influential segments in large road network and then imputing any missing measurements in the selected segments using Kriging.

The rest of the paper is organised as follows. Section 2 discusses some related work followed by the presentation of traffic flow and measurement model used in this work in Section 3. A background theory of random sets, covariance and variograms, which is the building block of Kriging is presented in Section 4. Recursive Bayesian estimation and particle filters (PF) are presented in Section 5. The proposed method was discussed in Section 5.3. Results and discussion are presented in Section 6.2 with conclusions being drawn in Section 7.

## 2. Related Work

A review of three different missing data imputation methods was presented by Ref. [27]. These include interpolation, prediction and statistical learning. The interpolation method imputes missing measurement at the particular location by averaging all historical measurements at that location at similar times of day. Prediction methods use a deterministic mathematical description to model the relationship between historical and future data. The statistical methods on the other hand treats the traffic as a random variable and tries to capture stochastic nature of the traffic pattern into the imputation algorithm.

In Refs. [28,29,30], a multi-resolution approximation using linear combinations of basis functions was proposed to address computational complexity of large datasets. The novelty in Ref. [28] lies in the use of multiple basis functions computed at lower resolutions closer to observation locations and then combining them to capture the different covariance functions with varying properties. The solution is achieved by dividing the spatial domain recursively into small regions and smaller sub-regions until the fine-scale dependencies are captured.

Nychka et al. [28] used radial basis functions (RBF) and special type of Gaussian Markov random field (GMRF) called spatial autoregressive (SAR) model to model the spatial correlation among the coefficients while Katzfuss et al. [30] automatically determines the appropriate basis/covariance function. Whereas in Ref. [29], it was assumed that the sub-domains are independent, Ref. [30] assumed depended sub-domains and performed full-scale approximation. As the computations are done locally in parallel, it is possible to fuse multisensor data sources, in which case, the different covariance functions are used for each data source. While both mentioned that the approach could be extended to non-stationary functions, it was not implemented nor was there a derivation for such. The different methods on missing traffic data imputation considered small road network in the range of a few kilometres.

## 3. Model Formulation

Consider a general discrete time state space system of a form,

(1) $$x_{k+1}=f_{k}\left(x_{k}\right)+v_{k}\;\mathrm{or}\;p\left(x_{k+1}|x_{k}\right),$$

(2) $$z_{k}=h_{k}\left(x_{k}\right)+w_{k}\;\mathrm{or}\;p\left(z_{k}|x_{k}\right),$$

where $f_{k}$ is a nonlinear function of the target state vector $x_{k}$, $h_{k}$ represents a nonlinear relationship between sensor output $z_{k}$ and target state vector $x_{k}$ affected by a measurement noise $w_{k}$. In addition, $p(x_{k+1}|x_{k})$ is the probability density function of the new state $x_{k+1}$ given the previous state $x_{k}$, and $p(z_{k}|x_{k})$ is the likelihood function of the measurement $z_{k}$ given the state $x_{k}$.

### 3.1. Stochastic Compositional Traffic Flow Model

In this work, a stochastic compositional model (SCM) [31] is considered for modelling of a motorway/freeway vehicle traffic evolution expressed as $p(x_{k+1}|x_{k})$ in Equation (1). This extended cell-transition model incorporates traffic speed and uses forward and backward waves to describe the complex relationship between traffic behaviour especially for a large road network. SCM utilises sending and receiving functions which models the stochastic nature of traffic state evolution. The vehicles that are able to leave a cell are represented by receiving functions while those that are allowed to enter a cell are determined by the receiving functions. The receiving functions are usually less than or equal to the sending functions to obey the law of conservation of vehicles.

**Figure 1:**
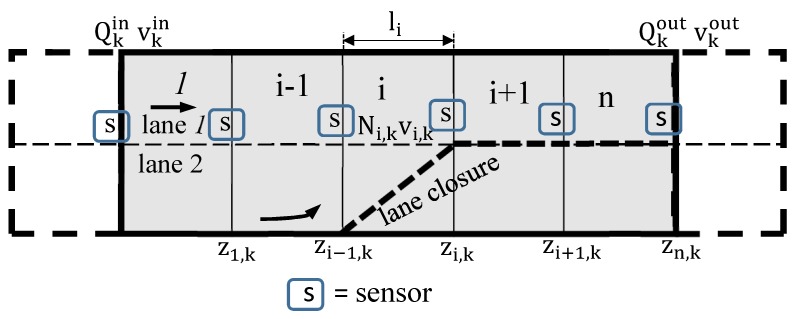
Stochastic compositional model (SCM) road network showing segments and measurement points [31].

In the SCM, the road network is divided into a given number of cells, *n*, also called segments. Each segment has a length $L_{i}$ and number of lanes $l_{i}$ as shown in Figure 1, where i=(1,⋯,n). At any given time period *k*, a certain number of vehicles $Q_{i,k}$ crosses the boundary between two segments *i* and i+1. The number of vehicles in a given cell *i* within the same time period *k* is represented by $N_{i,k}$ with their average speed given by $v_{i,k}$.

The overall state vector at time *k* is given by $x_{k}={\left[x_{1,k}^{T},x_{2,k}^{T},\cdots ,x_{n,k}^{T}\right]}^{T}$ where $x_{i,k}={\left[N_{i,k},v_{i,k}\right]}^{T}$ is the local state vector at segment *i*. Equations (3)–(5) models traffic state evolution within the cells.

(3) $$x_{1,k+1}=f_{1}\left(Q_{k}^{in},v_{k}^{in},\;x_{1,k},\;x_{2,k},\;\eta_{1,k}\right)$$

(4) $$x_{i,k+1}=f_{i}\left(x_{i-1,k},\;x_{i,k},\;x_{i+1,k},\;\eta_{i,k}\right)$$

(5) $$x_{n,k+1}=f_{n}\left(x_{n-1,k},\;x_{n,k},\;Q_{k}^{out},\;v_{k}^{out},\;\eta_{n,k}\right)$$

where $Q_{k}^{in}$, called inflow represents the number of vehicles entering the first cell with average speed $v_{k}^{in}$, $Q_{k}^{out}$, called outflow represents the number of vehicles leaving the last segment with average speed $v_{k}^{out}$. The inflow and outflow vehicles with their average speeds are called the boundary conditions and are supplied to the model at the beginning.

### 3.2. Measurement Model

Consider the road network shown in Figure 1 divided into different segments with *n* boundaries. The traffic state at a boundary j∈J=1,2,…,n is sampled at discrete time steps $t_{s}$, s=1,2,…, to give $z_{j,s}={\left(Q_{j,s},v_{j,s}\right)}^{T}$. The measurements at all the boundaries are collected into a matrix given by $Z_{s}={\left(z_{1,s}^{T},z_{2,s}^{T},\ldots ,z_{n,s}^{T}\right)}^{T}$. The relationship between the sampling interval $\Delta t_{s}$ and the state update time step $\Delta t_{k}$ Equation (1) is such that sampling interval is split into *q* state update time steps. That is, $\Delta t_{s}=q\Delta t_{k}$. The measurement model and noise is represented by Equation (6) with error $\xi_{s}$ = ${\left(\xi_{Q_{j,s}},\xi_{v_{j,s}}\right)}^{T}$. With the assumption of Gaussian noise, $z_{j,s}$ can be expressed as:

(6) $$z_{j,s}=\left(\begin{array}{c} Q_{j,s}\\ v_{j,s} \end{array}\right)+\xi_{s}.$$

## 4. Random Sets, Covariances and Variograms

### 4.1. Random Set

Let z(r) be a non-stationary set of *m* measurements $z\left(r\right)={\left[z\left(r_{1}\right),z\left(r_{2}\right),\cdots ,z\left(r_{n}\right)\right]}^{T}$, in our case average vehicle speeds v(r) or vehicle counts N(r), observed in segments i=1,⋯,n at locations $r_{i}=\left(r_{1},r_{2},\cdots ,r_{n}\right)$, where $r_{i}={\left(r_{i}^{x},r_{i}^{z}\right)}^{T}$. Note that location of each sensor is uniquely defined by the segment topology (Figure 1). Then, according to Equation (7),

(7) $$z\left(r\right)∼N\left(\mu (r),K(r)\right)$$

the random set of variables z(r) can be approximated by a Gaussian process which is uniquely defined by the drift, i.e., the mean field μ(r)=E[z(r)], and the corresponding covariance $K\left(r^{T},r\right)\;=\;{\left(z(r)\;-\;\mu (r)\right)}^{T}\left(z(r)-\mu (r)\right)$. Individual samples $z(r_{i})$ of the set Equation (7) can be also expressed as

(8) $$z\left(r_{i}\right)=\mu \left(r_{i}\right)+\delta \left(r_{i}\right)$$

with drift $\mu (r_{i})$ and residual $\delta (r_{i})$. Based on the degree to which the moments, in our case mean and variance of the random set z(r) are dependent (or independent) on a spatial relationship between points $r_{i}=\left(r_{1},r_{2},\cdots ,r_{n}\right)$, the 1st or the 2nd-order of stationarity can be recognised. While, the 1st-order stationary random set assumes a constant mean E[z(r)]=μ(r)=μ, the 2nd-order stationary random field assumes a linear drift between the increments $E\left[z\left(r\right)\right]=\mu \left(r\right)=\sum_{n=0}^{N}\alpha_{n}f\left(r\right)$. In the rest of this sequel, we assume that the random set z(r) is *intrinsic* and stationary on the 2nd-order.

### 4.2. Covariance

By assuming that random variables z(r) are 2nd-order stationary and isotropic, the covariance function $K(r_{i},r_{j})$ in Equation (7) reads as follows:

(9) $$\begin{array}{cc} K(r_{i},r_{j}) & =E\left[{\left(z\left(r_{i}\right)-\mu \right)}^{T}\left(z\left(r_{j}\right)-\mu \right)\right],\\ K(r_{i},r_{j}) & =E\left[{\left(z\left(r_{i}\right)-\mu \right)}^{T}\left(z\left(r_{i}+h\right)-\mu \right)\right]. \end{array}$$

The role of covariance function $K(r_{i},r_{j})$ is to model the correlation between measurements $z(r_{i})$ and $z(r_{j})$ observed at locations $r_{i}$ and $r_{j}$ based on their separation distance called a *lagh*. Since the correlation between two random variables solely depends on their spatial distance, and not at all on their location, the *lag* can be conveniently expressed as an Euclidean $l_{2}$ norm, defined as $h=\left|\right|r_{j}-r_{i}{\left|\right|}_{2}$. Above statements can be summarised into the following equalities, i.e., the isotropy assumptions:

(10) $$\begin{array}{cc} h=r_{j}-r_{i} & \Rightarrow {\left||h|\right|}_{2}=h\Rightarrow \\ K(r_{i},r_{j})= & K\left(r_{i},r_{i}+h\right)=K\left(h\right). \end{array}$$

For a straight stretch of motorway (Figure 1) this is equivalent to the path length through the road network.

**Figure 2:**
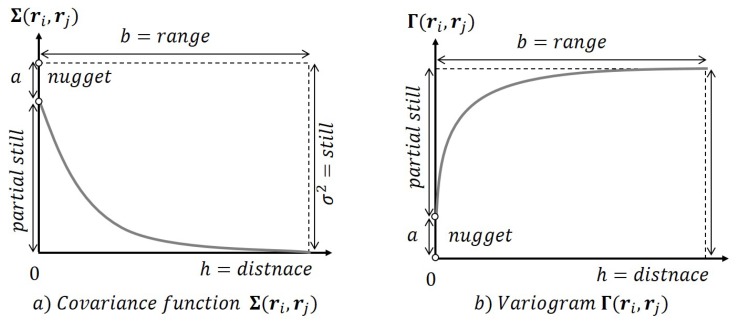
Covariance and variogram models.

The process of covariance function $K(r_{i},r_{j})$ modelling requires to find a covariance curve that has the best fit to the empirical data Equation (9), possibly being a subject to constraints. The covariance models to choose from include exponential, spherical, Gaussian, linear or power model [14]. In this work, the best fit for the dataset was achieved by the exponential model depicted in Figure 2a and given by

(11) $$K\left(r_{i},r_{j}\right)=\left\{\begin{array}{cc} c-a & if|r_{j}-r_{i}|=0,\\ \left(c-a\right)e^{-\frac{3|r_{j}-r_{i}|}{b}}\} & if|r_{j}-r_{i}|>0, \end{array}\right.$$

where *a* is the nugget, *b* is the range and *c* is still.

As can be observed in Figure 2a, observe that the covariance function decreases with distance, so it can be thought of as a similarity function.

## 5. Recursive Bayesian Estimation

### 5.1. Bayesian Estimation

Consider a general discrete time state space system represented as in Equations (1) and (2). The goal of Bayesian estimation is to infer the state variable $x_{k}$ as defined in Section 3.1 with the available sensor measurements $z_{1:k}$. By using the Bayesian framework, this estimation problem relates to the recursive evaluation of the probability density function (PDF) $p(x_{k}|z_{1:k})$ in two consecutive steps, the prediction and the measurement update of the state vectors.

(12) $$p\left(x_{k-1}|z_{1:k-1}\right)\overset{\mathrm{Prediction}}{\underset{\mathrm{Update}}{\to }}p\left(x_{k}|z_{1:k-1}\right)$$

(13) $$p\left(x_{k}|z_{1:k-1}\right)\overset{\mathrm{Measurement}}{\underset{\mathrm{Update}}{\to }}p\left(x_{k}|z_{1:k}\right)$$

The prediction state density $p(x_{k}|z_{1:k-1})$ of state $x_{k}$ is calculated from the prior PDF $p(x_{k-1}|z_{1:k-1})$ by using Chapman-Kolmogorov equation

(14) $$p\left(x_{k}|z_{1:k-1}\right)=\int p\left(x_{k}|x_{k-1}\right)p\left(x_{k-1}|z_{1:k-1}\right)dx_{k-1}.$$

Equation (14) follows the 1st order Markov property which assumes that $p(x_{k}|z_{1:k-1})$ only depends on state $x_{k}$ and $x_{k-1}$ at time *k* and k−1 respectively. The measurement update $p(x_{k}|z_{1:k})$ is computed from the prior distribution of Equation (14) and measurements $z_{1:k}$ by a Bayesian formula which results in

(15) $$p\left(x_{k}|z_{1:k}\right)=\frac{p\left(z_{k}|x_{k}\right)p\left(x_{k}|z_{1:k-1}\right)}{p(z_{k}|z_{1:k-1})}$$

The 1st order Markov property for Equation (15) implies that $p(x_{k}|z_{1:k})$ only depends on measurement $z_{k}$ at time *k*.

### 5.2. Particle Filter

Arguably, the most popular algorithm for nonlinear recursive estimation is the particle filter (PF), extensively evaluated in Ref. [32]. PF represents any arbitrary probability density function $p(x_{k}|z_{1:k})$ by samples or particles $x_{k}^{l}$, where $l=1,\cdots ,N_{p}$ is the number of particles, i.e.,

(16) $$x_{k}^{l}\approx p\left(x_{k}|z_{1:k}\right),$$

The particles are used to form an approximative distribution as

(17) $$p\left(x_{k}|z_{1:k}\right)\approx \hat{p}\left(x_{k}|z_{1:k}\right)=\sum_{i=1}^{N}w_{k|k}^{l}\delta \left(x_{k}-x_{k}^{l}\right),$$

where $\hat{p}\left(x_{k}|z_{1:k}\right)$ is an approximated distribution, $\delta (x_{k}-x_{k}^{l})$ is a the Dirac delta function and $w_{k|k}^{l}$ the weights of the particles satisfying $\sum_{l=1}^{N_{p}}w_{k|k}=1$. The time update of the Bayesian recursion Equation (1) is in case of PF evaluated as

(18) $$\begin{array}{cc} p(x_{k} & |z_{1:k-1})\\ \approx & \int p\left(x_{k}|x_{k-1}\right)\sum_{l=1}^{N}w_{k-1|k-1}^{l}\delta \left(x_{k-1}-x_{k-1}^{l}\right){\mathrm{dx}}_{k-1},\\ \approx & \sum_{l=1}^{N}w_{k-1|k-1}^{l}\int p\left(x_{k}|x_{k-1}\right)\delta \left(x_{k-1}-x_{k-1}^{l}\right){\mathrm{dx}}_{k-1},\\ \approx & \sum_{l=1}^{N}w_{k-1|k-1}^{l}p\left(x_{k}|x_{k-1}\right). \end{array}$$

The particles $x_{k-1}^{l}$ in above Equation (18) are sampled from proposal distribution $\pi (x_{k}|x_{k-1}^{l})$, i.e., $x_{k-1}^{l}\approx \pi \left(x_{k}|x_{k-1}^{l}\right)$. Proposal distribution is very often defined by the state transition PDF, that is $\pi \left(x_{k}|x_{k-1}^{l}\right)=p\left(x_{k}|x_{k-1}^{i}\right)$. In this case, the weights updates result to

(19) $$\begin{array}{cc} w_{k|k-1}^{l}= & \frac{p(x_{k}|x_{k-1}^{l})}{\pi (x_{k}|x_{k-1}^{l},z_{k})}w_{k-1|k-1}^{l},\\ = & \frac{p(x_{k}|x_{k-1}^{l})}{p(x_{k}|x_{k-1}^{l})}w_{k-1|k-1}^{l}=w_{k-1|k-1}^{l}. \end{array}$$

The measurement update $p(x_{k}|z_{1:k})$ (2) is computed by a Equation (15), which can be in terms of the particles $x_{k}^{l}$ represented as

(20) $$\begin{array}{cc} p(x_{k}|z_{1:k})\propto & p\left(z_{k}|x_{k}\right)p\left(x_{k}|z_{1:k-1}\right),\\ \approx & \sum_{l=1}^{N_{p}}w_{k|k-1}^{l}p\left(z_{k}|x_{k}\right)\delta \left(x_{k}-x_{k}^{l}\right). \end{array}$$

Similarly, the particle filter weights are updated as

(21) $$w_{k|k}^{l}=\frac{w_{k|k-1}^{l}p(z_{k}|x_{k}^{l})}{\sum_{l^{{}^{\prime }}=1}^{N_{p}}w_{k|k-1}^{l^{{}^{\prime }}}p(z_{k}|x_{k}^{l^{{}^{\prime }}})}.$$

The denominator in Equations (15) and (21) is only a normalising factor independent of $x_{k}$ thus can be safety omitted if the distribution is numerically normed as shown by Equations (20) and (21).

(22) $$\begin{array}{cc} w_{k|k}^{l}\propto & w_{k|k-1}^{l}p(z_{k}|x_{k}^{l}),\\ \approx & w_{k|k-1}^{l}p(z_{k}|x_{k}^{l}). \end{array}$$

The MC recursion tempt to degrade over time as all relative weights would tend to zero except for one that tends to one. Therefore, when particle depletion ratio reaches 0.5 a Sampling Importance Resampling (SIR) or Sampling Importance Sampling (SIS) techniques are applied in the recursion.

### 5.3. Missing Measurement Interpolation and Improved Likelihood Computation

For a large road network with many segments, using all the measurements in the particle filter measurement update step becomes computationally intensive. Column based matrix decomposition approach similar (as earlier stated in Section 1) to the work of [26] is employed to select the most probable segments that would give acceptable accuracy.

The idea is to select *m* most influential segments from all available segments *n* using CBMD and then estimating missing measurements (if any) of the most influential segments using Kriging for improved particle likelihood computation. Let $Z_{n}\in R^{k\;\times \;n}$ represent a set of all segments or measurement locations in a given time period, where the rows, *k* is number of time instances at which the measurements were taken and columns *n* number of road segments.

The goal of CBMD is to approximate the measuremtns $Z_{n}$ with a subset of measurements $Z_{m}\;\in \;R^{k\;\times \;m}$ where m<n is a subset of the measurements using singular value decomposition (SVD) as Ref. [33]:

(23) $$Z_{m}=Z_{n}\Phi$$

where $\Phi \in R^{n\;\times \;m}$ is the transformation matrix that expresses every column of all measurement $Z_{n}$ in terms of the basis in $Z_{m}$. Having computed the SVD, the right singular matrix is used to assign a probability $P_{z_{i}}$ to each selected location according to:

(24) $$P_{z_{i}}=\frac{1}{r}\sum_{j=1}^{r}v_{i,j}^{2},i=1,...,n$$

where $v_{i,j}$ is the *i*th element of the *j*th right singular vector and *r* is the rank of the matrix. From the probabilities computed, *m* locations with the highest probabilities are chosen as the reduced measurement to approximate the entire network which is used in computing the particle filter likelihood during the measurement update step.

The likelihood function term $p(z_{k}|x_{k})$ in Equation (21), is computed when a new measurement arrives. The performance of the PF degrades substantially when there is a missing measurement. For the multivariate Gaussian distribution, the PDF is given by:

(25) $$p\left(z_{k}|x_{k}\right)=\frac{1}{\sqrt{2\pi }\left|R\right|}e^{-0.5\nu R^{-1}\nu^{T}},$$

where R is the covariance matrix of the measurement data, |R|≡det(R) is the determinant of R and ν is the difference between the PF predicted value (${\bar{z}}_{s}$) and measurement ($z_{s}$), given by:

(26) $$\nu =z_{s}-{\bar{z}}_{s}.$$

The measurement matrix $z_{s}$ can be expressed as,

(27) $$z_{s}=\left\{\begin{array}{cc} z_{s}^{meas} & \mathrm{measurement}\;\mathrm{from}\;\mathrm{sensor};\\ {\hat{z}}_{s}^{krig} & \mathrm{estimated}\;\mathrm{by}\;\mathrm{Kriging}. \end{array}\right.$$

where ${\hat{z}}_{s}^{krig}$ represents the value estimated by Kriging (when measurement is not available). Note that the sampling time index $t_{s}$ is split into *q* update time indices $t_{k}$ as mentioned in Section 3.2. The measurement update state of the PF is performed only when $t_{k}$≡$t_{s}$. The modified particle method is presented in Algorithm 1.

**Algorithm 1** Particle Filter for Traffic State Estimation with Kriging Estimated Measurements [24]
Road network approximationUse compressed sensing to select *m* most significant locations out of the *n* segments to be used for the measurement update step as defined in Section 5.3.InitialisationAt k=0; define all boundary conditions: number of samples, weight of samples as below,For $l=1,\cdots N_{p}$, $N_{p}$ number of particles;generate $N_{p}$ samples $\left\{x_{0}^{\left(l\right)}\right\}$ from the initial distribution $p(x_{0})$initialise the particle weights $w_{0}^{\left(l\right)}=\frac{1}{N_{p}}$.End forStart the iteration for k=1,2,⋯(a)Prediction stageFor $l=1,...,N_{p}$,sample $x_{k}^{\left(l\right)}∼p\left(x_{k}|x_{k-1}^{\left(l\right)}\right)$ according to SCM model equationsEnd for(b)Measurement Update:This step is performed when the sampling time $t_{s}$ equals the iteration count $t_{k}$ as defined in Section 3.2i. Estimate missing measurements in the *m* most significant locations with Kriging using Equations (28) and (33)ii. Compute the likelihoodsBased on Equation (6) compute the likelihood, $p(z_{s}|x_{s}^{\left(l\right)})$ of the particles using Equations (25)–(27)iii. Update the weights of the particles using the likelihood $p(z_{s}|x_{s}^{\left(i\right)})$ calculated from Equation (6)For $l=1,...,N_{p}$$\omega_{s}^{\left(l\right)}=\omega_{s-1}^{\left(l\right)}p\left(z_{s}|x_{s}^{\left(l\right)}\right)$End Foriv. Normalise the weights: ${\hat{\omega }}_{s}^{\left(l\right)}=\frac{\omega_{s}^{\left(l\right)}}{\sum_{l=1}^{N_{p}}\omega_{s}^{\left(l\right)}}$.(c)Update the predicted states (Output): ${\hat{x}}_{s}=\sum_{l=1}^{N_{p}}{\hat{\omega }}_{s}^{\left(l\right)}x_{s}^{\left(l\right)}$(d)Re-sample the weights (Selection) only when $t_{k}$ = $t_{s}$


### 5.4. Missing Data Estimation via Kriging Models

The underlying idea of Kriging is to obtain the response $z(r_{u})$, interpreted as a random variable positioned at the location $r_{u}$, by interpolating random variables $z\left(r\right)={\left[z\left(r_{1}\right),z\left(r_{2}\right),\cdots ,z\left(r_{n}\right)\right]}^{T}$ from Equation (7), i.e., observations $z(r_{i})$, i=1,⋯,n at locations $r_{i}$. The Kriging predictor incorporates the covariance structure among the observation points $z(r_{i})$ into the weights $w\left(r_{u}\right)\;=\;{\left[w_{1}\left(r_{u}\right),w_{2}\left(r_{u}\right),\cdots ,w_{n}\left(r_{u}\right)\right]}^{T}$ for predicting $\hat{z}\left(r_{u}\right)$ as a linear combination:

(28) $$\hat{z}\left(r_{u}\right)=\sum_{i=1}^{n}w_{i}\left(r_{u}\right)z\left(r_{i}\right)=w^{T}\left(r_{u}\right)z\left(r\right).$$

In order to assess the accuracy of the Kriging prediction $\hat{z}\left(r_{u}\right)$ w.r.t the real (true) value $z(r_{u})$ an error $\epsilon (r_{u})$ is declared

(29) $$\epsilon \left(r_{u}\right)=z\left(r_{u}\right)-\hat{z}\left(r_{u}\right).$$

The following criteria, evaluated in terms of mean $E\left[\epsilon (r_{u})\right]$ and variance $\mathrm{Var}\left[\epsilon (r_{u})\right]=\sigma_{\epsilon }^{2}\left(r_{u}\right)$ of the prediction error $\epsilon (r_{u})$ Equation (29), apply to any type of Kriging interpolation:

- Lack of bias, implies that $E\left[\epsilon (r_{u})\right]=0$ and therefore $E\left[\hat{z}\left(r_{u}\right)\right]=E\left[z(r_{u})\right]$. Thus, the Kriging interpolation is said to be the globally unbiased Equation (30).
  (30) $$E[z\left(r_{u}\right)-\hat{z}\left(r_{u}\right)]=0$$
- Minimum variance, implies that the mean of the squared deviations $\mathrm{Var}\left[\epsilon \left(r_{u}\right)\right]=\sigma_{\epsilon }^{2}\left(r_{u}\right)$ must be the minimal Equation (31),
  (31) $$\min_{1^{T}w=1}\mathrm{Var}\left[z\left(r_{u}\right)-\hat{z}\left(r_{u}\right)\right]\;\mathrm{or}\;\min_{1^{T}w=1}\sigma_{\epsilon }^{2}\left(r_{u}\right),$$
  subject to
  (32) $$\sum_{i=1}^{m}w_{i}=1^{T}w=1$$

Kriging weights $w(r_{u})$ are chosen such that the mean squared prediction error $\sigma_{\epsilon }^{2}\left(r_{u}\right)$ Equation (31), also known as Kriging variance or Kriging error is minimised as $\min_{1^{T}w=1}\sigma_{\epsilon }^{2}\left(r_{u}\right)$ over all $z(r_{u})$ Equation (31) subject to the unbiased conditions of Equations (30) and (32) by a Lagrange multiplier 2λ to give,

(33) $$w\left(r_{u}\right)=K{\left(r_{i},r_{j}\right)}^{-1}K\left(r_{i},r_{u}\right)-\mu 1.$$

where matrix $K(r_{i},r_{j})$ is the covariance between the individual samples and column vector $k(r_{i},r_{u})$ is a covariance function between samples and the point to interpolate.

## 6. Performance Evaluation

A road network with 1000 segments was simulated using SUMO software [34] to validate the proposed method. The segments are spaced 0.5 km apart and measurements (number of vehicles crossing each segment boundary with their average speed) taken every second. Traffic signs were installed at some locations to model the effect of congestion.

This is an extension of the previous work [25] where a smaller number segments was considered. The aggregate traffic flow and speed were sampled every 60 s and the results collected over a period of 10,800 s (3 h). Two types of vehicles, bus and passenger car was defined with the parameters as in Table 1.

The vehicles were added randomly into the network through the inflow boundary every one second and they travel through the network until they get to the last boundary when they leave the network. As a vehicle crosses each induction loop, it is counted with its speed. The average speed of the vehicles arriving at an induction loop over a period is recorded as the average speed. The entire statistics, flow, occupancy, and speed are collected in an output file for further processing.

### 6.1. Simulation Design

**Figure 3:**
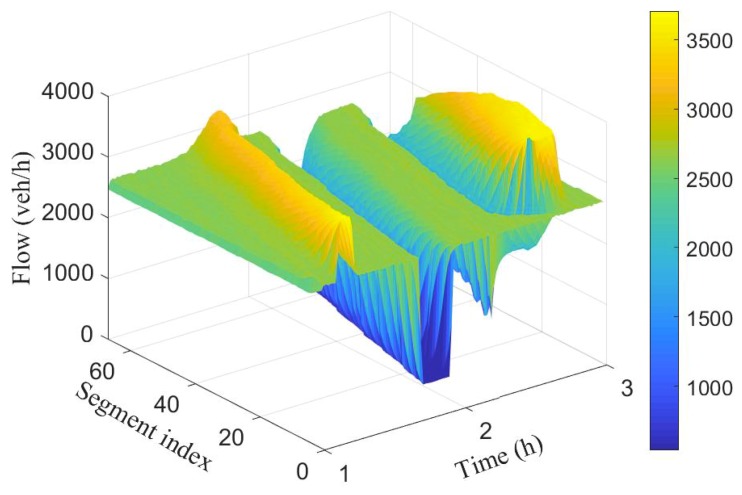
Spatio-temporal evolution of traffic flow for the 100 segment.

**Figure 4:**
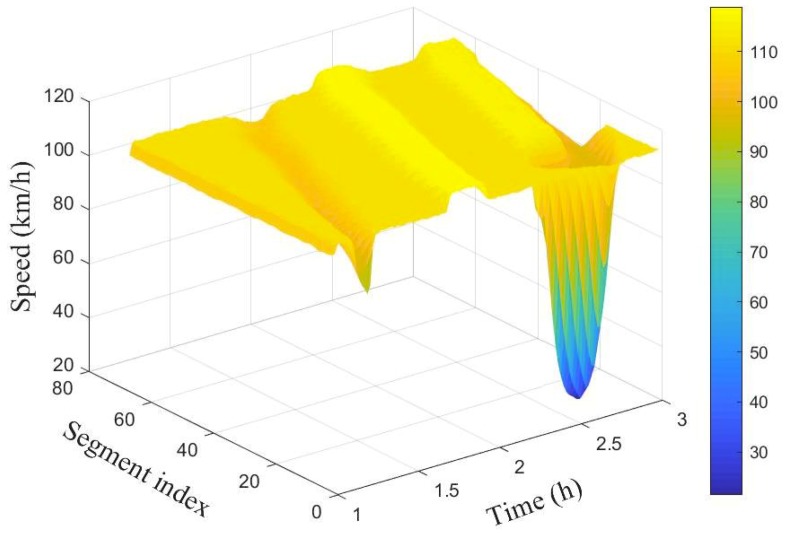
Spatio-temporal evolution of traffic speed for the 100 segment.

To simulate different scenarios such as congestion and free flow, (i) the number of lanes were decreased from 3 to 2, and (ii) the rate of vehicle injection into the network is varied at different time periods. Figure 3 and Figure 4 show the spatio-temporal evolution of the traffic and their corresponding average speed, respectively, for a 100 segment section. The average speed of the vehicles varies around 100 km/h when the flow was around 2000 veh/h. Between time interval [1.5 h, 1.7 h], the flow was increased slightly to cause congestion, this resulted to a decrease in the average speed as can be seen in the first spike from Figure 4.

Observe that the effect was felt more close to the inflow boundary. The vehicles’ speed increases marginally as they move into the network. Between time interval [1.6 h, 1.9 h] the flow was decreased leading to increase in the average speed. Finally, the number of lanes in segments 10 to 14 were reduced from 3 to 2 between time interval [2.4 h, 2.4 h] while maintaining vehicle injection rate. This results to substantive decrease in speed as can be seen (cone-shaped) in Figure 4. As the vehicles leave the segments with closed lanes, there is increase in their speed again.

### 6.2. Results and Discussion

In order to test the prediction accuracy for different levels of sparsity, a statistical measure namely the root mean squared error (RMSE), was computed. Note, $z_{i}$ is the ground truth or actual measurement, ${\hat{z}}_{i}$ is the estimated value and $m_{r}$ is number of independent Monte Carlo runs. The measurements at some boundaries were randomly removed each time and then estimated using the proposed method. Different missing data rates (from 10, 20, …, 70%) were investigated by randomly removing measurements at some locations using leave one out cross validation. This was repeated for 100 Monte Carlo runs and the average value used.

(34) $$RMSE=\sqrt{\frac{1}{m_{r}}\sum_{i=1}^{m_{r}}{\left[z_{i}-{\hat{z}}_{i}\right]}^{2}}.$$

**Figure 5:**
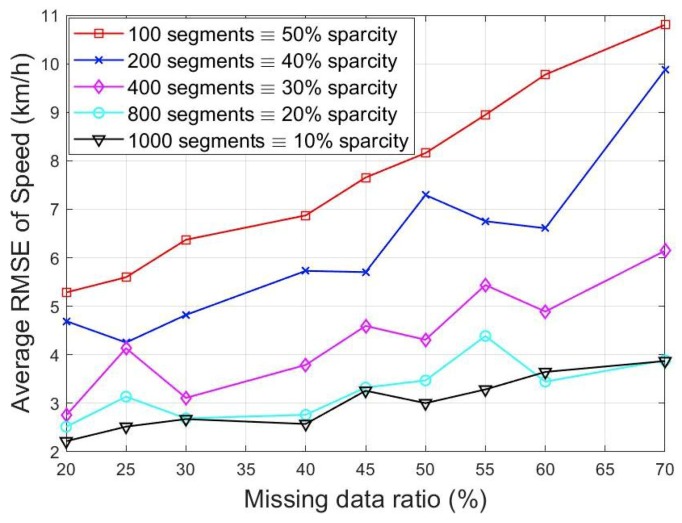
RMSE of speed at different missing data ratios.

**Figure 6:**
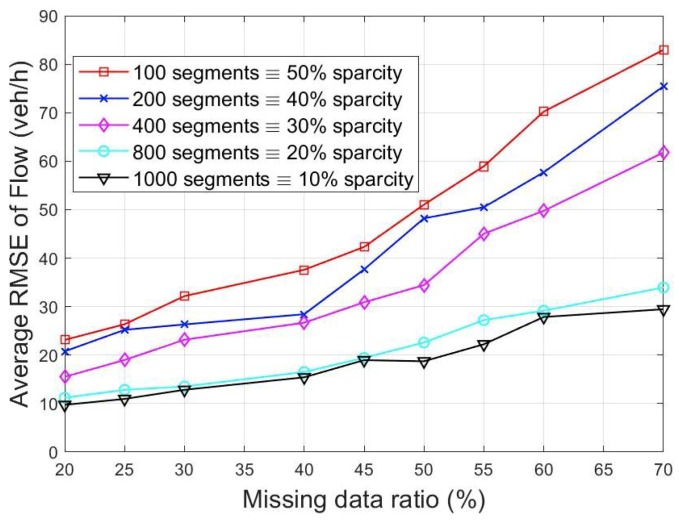
RMSE of flow at different missing data ratios.

Figure 5 and Figure 6 show the average estimation error for the different missing data ratios and the number of segments. It would be observed that the prediction error increases with the missing data rate. This is expected as less data is available for the computation. This effect could be reduced further by incorporating a mechanism known as multi resolution approach [30].

The plots also show that the higher the number of segments used, the better the accuracy. This could be attributed to the fact that there is better information exchange within the network and hence, on average, more segments with available data are used for the higher dimensional segments scenario. This is in agreement with [35] where estimation accuracy in the presence of sparse sensor data was improved by exchanging particle weights between segments. For instance, a 10 segment network with 70% missing data ratio will result to using only 3 data points to estimate the remaining 7 missing locations. The chances of these 3 locations correlating with the other 7 is lower compared to when 30 out of 100 data points are available.

**Figure 7:**
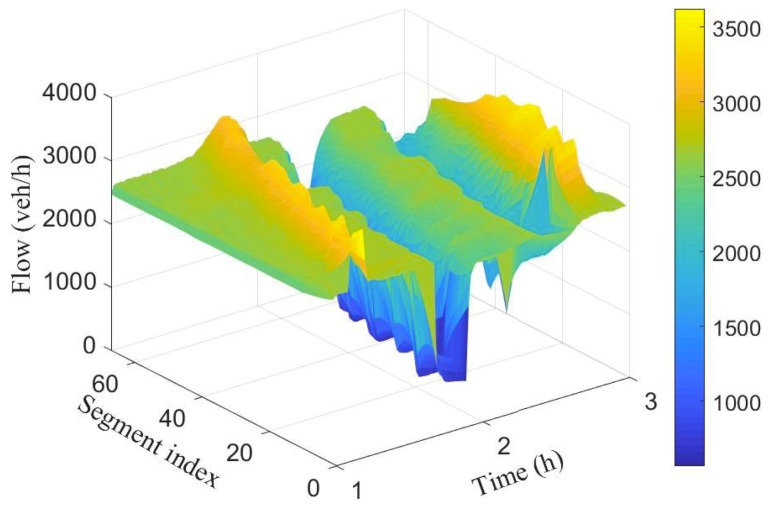
Estimated flow for the 100 segment with 30% missing data.

**Figure 8:**
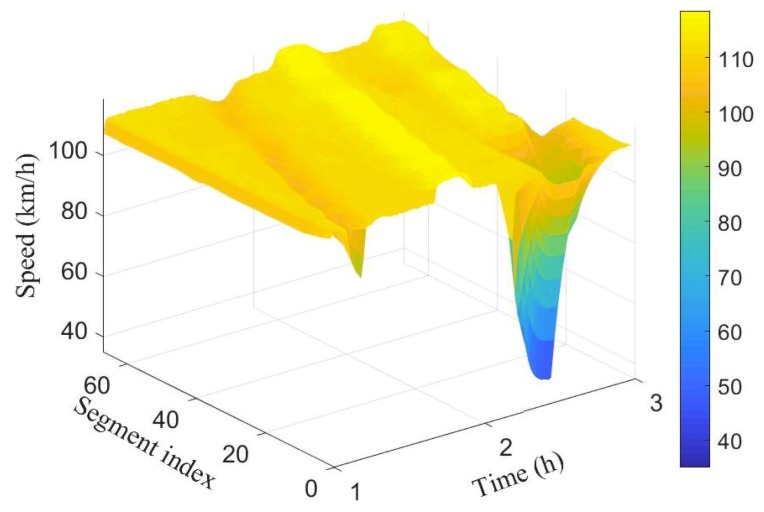
Estimated speed for the 100 segment with 30% missing data.

Figure 7 and Figure 8 show the spatio-temporal evolution of the traffic flow and the corresponding average speed for the 100 segment scenario.

The number of vehicles crossing each boundary in space and their associated average speed is represented by the colour bar.Compared to the ground truth shown in Figure 3 and Figure 4, it is evident that estimated number of vehicles crossing segment boundaries and the associated speeds have been estimated with a good accuracy. Observe also that the estimated flow and speed captured the periods where there is drop in number of vehicles and decrease in speed.

## 7. Conclusions

This paper presented a traffic estimation for a large road network with different missing data ratios. The computational overhead of the large network was addressed by using a method called reduced measurement space proposed by [26] to select the most influential and information rich segments in the road network. These are subsequently used in the particle filter measurement update step. Missing data in the selected segments are imputed using Kriring. A 1000-segment road network was simulated using SUMO. Different missing data ratios ranging from 10% to 70% were tested for different sizes of road network ranging from 100 to 1000 segments.

The results indicate that considering a larger number of segments would reduce the overall estimation error even when the missing data ration is high. From the foregoing results and discussion, it is recommended that the best estimation accuracy would be obtained when the entire road network is considered at once. The effects of computational overhead could further be reduced by using a distributed approach with a central control unit.

## Figures and Tables

**Table 1:** SUMO simulation parameters.

	Car	Bus
Max speed	25 m/s	20 m/s
Acceleration	1.0 m/s${}^{2}$	0.8 m/s${}^{2}$
Deceleration	4.5 m/s${}^{2}$	4.5 m/s${}^{2}$
Sigma (driver perfection)	0.5	0.5
Length	5 m	10 m
Minimum Separation	2.5 m	3 m
